# A Review of 3D Modalities Used for the Diagnosis of Scoliosis

**DOI:** 10.3390/tomography10080090

**Published:** 2024-08-02

**Authors:** Sampath Kumar, Bhaskar Awadhiya, Rahul Ratnakumar, Ananthakrishna Thalengala, Anu Shaju Areeckal, Yashwanth Nanjappa

**Affiliations:** Department of Electronics and Communication Engineering, Manipal Institute of Technology, Manipal Academy of Higher Education, Manipal 576104, India; kumar.sampath@manipal.edu (S.K.); awadhiya.bhaskar@manipal.edu (B.A.); rahul.ratnakumar@manipal.edu (R.R.); anantha.kt@manipal.edu (A.T.); anu.areeckal@manipal.edu (A.S.A.)

**Keywords:** idiopathic scoliosis, 3D deformity, 2D radiographs, computed tomography (CT), magnetic resonance imaging (MRI), radiation exposure

## Abstract

Spine radiographs in the standing position are the recommended standard for diagnosing idiopathic scoliosis. Though the deformity exists in 3D, its diagnosis is currently carried out with the help of 2D radiographs due to the unavailability of an efficient, low-cost 3D alternative. Computed tomography (CT) and magnetic resonance imaging (MRI) are not suitable in this case, as they are obtained in the supine position. Research on 3D modelling of scoliotic spine began with multiplanar radiographs and later moved on to biplanar radiographs and finally a single radiograph. Nonetheless, modern advances in diagnostic imaging have the potential to preserve image quality and decrease radiation exposure. They include the DIERS formetric scanner system, the EOS imaging system, and ultrasonography. This review article briefly explains the technology behind each of these methods. They are compared with the standard imaging techniques. The DIERS system and ultrasonography are radiation free but have limitations with respect to the quality of the 3D model obtained. There is a need for 3D imaging technology with less or zero radiation exposure and that can produce a quality 3D model for diseases like adolescent idiopathic scoliosis. Accurate 3D models are crucial in clinical practice for diagnosis, planning surgery, patient follow-up examinations, biomechanical applications, and computer-assisted surgery.

## 1. Introduction

Scoliosis is a common deformity of the human spine. Idiopathic scoliosis is common among the various types found in the pediatric population of the age group 10 to 18 [1,2]. Idiopathic scoliosis accounts for nearly 85% of all scoliosis cases registered. It is defined as the lateral curvature of the spine of more than 10^0^ in the frontal plane along with the vertebral rotation. Hence, it is very well characterized by three-dimensional (3D) modalities [3]. The deformities must be assessed in all three planes for proper diagnosis. Three-dimensional reconstruction of the spine is very essential in several studies. For example, in finding the effect of the treatment after wearing Boston braces, comparison of the spine is carried out in pre- and postoperative cases and the progression of scoliosis is determined in 3D. Computed tomography (CT) has limited application in scoliosis diagnosis, as it is carcinogenic and obtained in the supine position. The supine position changes the actual 3D spinal deformity [4]. For scoliosis diagnosis, a 3D model of the standing position gives accurate results [5]. Magnetic resonance imaging (MRI) is also not suitable, as the 3D model in this case is obtained in the supine position. If any screws, hooks, or rods are introduced into the subject’s body as a spine correction tool, an MRI cannot be taken [6]. Therefore, 3D reconstruction from one or more radiographs taken in different planes is the most popular and promising method for the diagnosis of scoliosis [7]. The DIERS formetric scanner system, the EOS imaging system, and ultrasonography techniques for 3D reconstruction of the spine are discussed. This article reviews the 3D evaluation techniques for the diagnosis of scoliosis and then discusses the latest methods and future directions.

## 2. 3D Reconstruction from Multiplanar Radiographs

**Figure 1:**
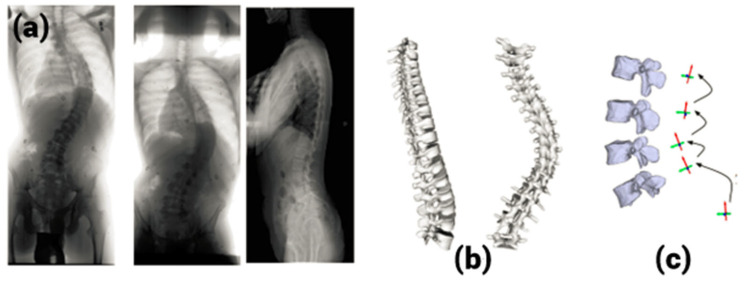
A 3D reconstruction from multiplanar radiographs. (**a**) Radiographs in three views, (**b**) 3D reconstruction, (**c**) 3D reconstruction of vertebrae.

The 3D reconstruction of human ribcages from multiplanar radiographs was the first attempt of a method other than CT or MRI modelling methods [8,9]. Dansereau et al. [9] came up with a method based on three radiograph views: a postero-anterior view and a postero-anterior view with 20° leaning incidence and a lateral view. Figure 1 shows the entire process. This technique was time-consuming, as manual digitization was needed twice (on each postero-anterior view). Eleven points on the rib midlines, including five radio-opaque markers, needed to be identified on the radiographs. Since the process of digitization is manual, the technique was slow and dependent on the operator. Further, the third radiograph was obtained with a non-standard format. Makino et al. [10] tried to reconstruct the scoliotic spine using multiplanar images obtained from a CT scan. This method has limitations like a risk of high radiation dosage and nonstandard image formats. Hence, the method did not become popular, and researchers tried to get 3D reconstruction from only two radiographs.

## 3. 3D Reconstruction from Biplanar Radiographs

Scoliotic patients normally undergo biplanar radiographs for the brace design and other follow-up examinations and treatment of the spine. These radiographs can be used for 3D reconstruction without exposing the patients to further radiation. Hence, it is a popular method. Three-dimensional reconstruction from biplanar radiographs is also known as stereo-radiographic reconstruction. Depth information is extracted from the biplanar radiographs with the help of a calibration device. Three-dimensional reconstruction from biplanar radiographs can be classified under the following categories: stereo-corresponding point-based methods [11,12,13,14], non-stereo-corresponding point-based methods [15,16,17,18], hybrid methods [19,20,21], surface topography [22,23,24], and low-dose radiography [25,26,27].

### 3.1. Stereo-Corresponding Point (SCP)-Based Methods

**Figure 2:**
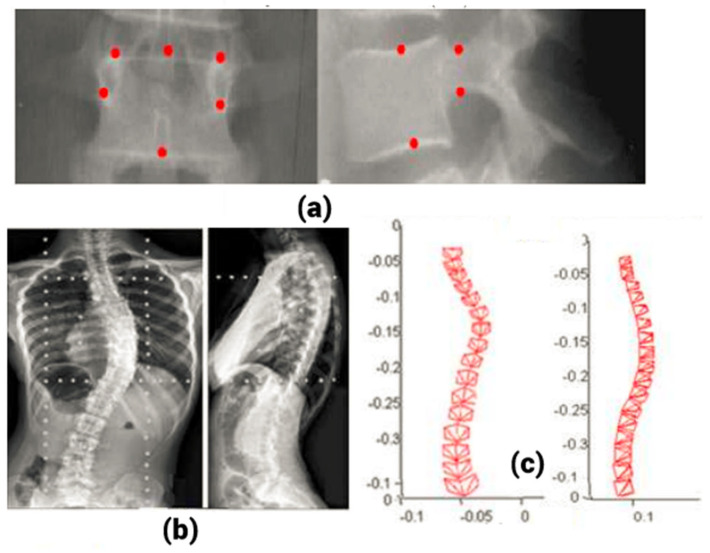
(**a**) Stereo-corresponding points on the vertebra used for SCP reconstruction, (**b**) sample biplanar radiographs, (**c**) SCP reconstruction [28]. SCP stereo corresponding point.

SCP-based methods [11] require calibration of the radiographic environment. Direct linear transformation (DLT) [12] was the algorithm used for this purpose, which requires a large calibration object. For intra-operative radiographs, it is not suitable. To overcome this difficulty, based on non-linear optimization, an explicit calibration algorithm that requires a small calibration object was used. Self-calibration techniques can also be used. For intra-operative radiographs, self-calibration needs to be combined with the landmark identification process for proper 3D reconstruction. For spine reconstruction, six anatomical landmarks per vertebra were chosen. They were the top and bottom of both pedicles and the top and bottom of the vertebral endplates. These were identified manually by an expert and named SCPs (Figure 2). The parameters obtained from the self-calibration technique were used for 3D spine reconstruction by triangulation [13]. This method is known as the SCP method. The main challenge of this method is the proper identification of at least six corresponding landmarks on the biplanar radiographs.

Stokes [14], Pearcy [29], Bernard et al. [30], and Andre et al. [31] used the SCP method for 3D spine reconstruction. Stokes [14] and Pearcy [29] obtained a 3D model by locating six to nine SCPs. The DLT algorithm was used for triangulation. They were not able to obtain a precise model with this process due to consideration of only local data compared to universal configuration of anatomical shapes. To accomplish more precise results, Aubin et al. [32] used a generic model attained from a 3D scanner to construct a morpho-realistic model. With the help of six SCPs as control points, this method uses the dual kriging algorithm [33] on the generic object to obtain the 3D model. For 3D reconstruction, the DLT algorithm was used. Although improved results were obtained, for better description of the vertebrae, there was a need for more landmark points. Gauvin et al. [34] used additional landmark points to achieve more accurate 3D models. They used this technique on the pelvis bone with 19 SCPs as control points. The DLT algorithm was used for the 3D reconstruction process. In this method, the average variation resulting from direct measurements was 4.8 mm, which was very high. Aubin et al. [32] also used the same technique for the vertebrae using six SCPs and 21 additional points. For the additional SCPs, the errors were 2.1 (mean) and ±1.5 mm (standard deviation) compared to the direct measurements obtained. Sampath Kumar et al. [35] have developed a new method known as combined SCP and geometric reconstruction (CSCPG) for 3D reconstruction of the spine. Along with the SCP reconstruction, some geometric parameters were extracted from the radiograph to obtain the 3D model. The method was faster and the reconstruction accuracy was within the acceptable limits. Though better results were obtained, there was inter-observer variability and intra-observer variability observed in the results. In addition, the identification of additional points was time-consuming. The drawbacks of these 3D reconstruction techniques are the ambiguity in stereo-corresponding point detection, repeatability issues, observer variability, and prolonged process. In conclusion, due to the limited number of SCPs detectible on radiographs, these techniques have low precision compared to other techniques.

### 3.2. Non Stereo-Corresponding Point-Based (NSCP) Methods

**Figure 3:**
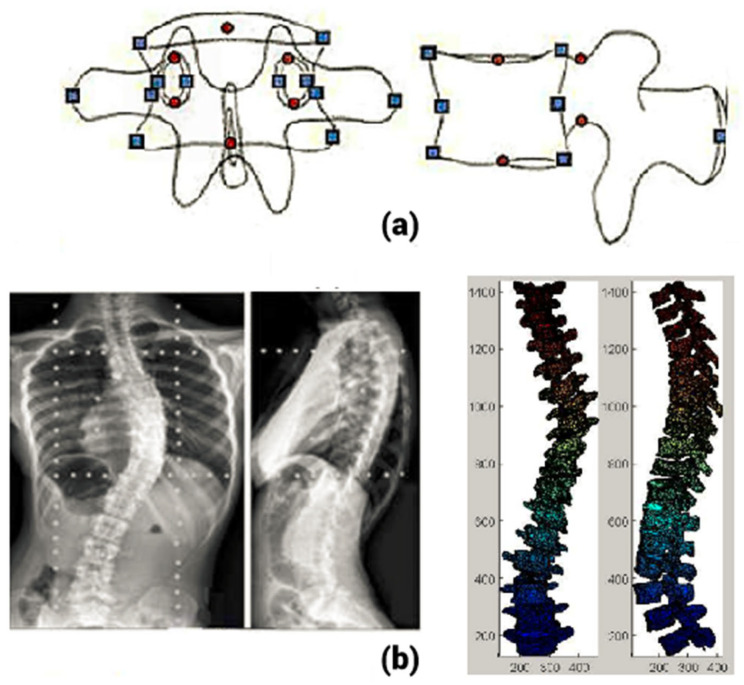
(**a**) Non-stereo-corresponding points on the vertebra used for NSCP reconstruction, (**b**) sample NSCP reconstruction [35]. NSCP—non-stereo-corresponding point.

Due to the limits of SCP-based techniques, NSCP-based methods were proposed. In this method, points visible only on one radiograph were used for 3D reconstruction. It is based on this theory that any non-stereo-corresponding point must lie on a line connecting the projection of the point in one view and the X-ray source. They are known as NSCP points (Figure 3).

They provide additional landmark points for the 3D reconstruction that can be detected in only one of the radiographs. The introduction of NSCPs allows for the 3D reconstruction of both SCPs and NSCPs, which in turn results in a more detailed and refined 3D geometrical model of the spine, with increased accuracy. In this technique, with respect to the SCPs and NSCPs on the radiographs, an elastic object is deformed to obtain the 3D spine. Mitton et al. [15] proposed an NSCP-based technique for 3D reconstruction of the human spine. The calibration of the radiological environment was performed by means of the known points on the calibration object. Later, the DLT algorithm was used for 3D reconstruction of SCPs. Then, 3D coordinates of the NSCPs were identified by defining the line joining the projection of an anatomical landmark point in the radiographs and the source. By defining a generic object, points on their lines in the radiograph are initialized. Using an optimization technique, the position of the points on their lines is identified. By considering the similarity in shape with the generic object, a 3D reconstruction of the NSCP point is achieved. Finally, with the help of dual kriging of the generic object with SCPs and NSCPs as control points, a morpho-realistic shape of the spine is obtained [36]. Mitton et al. [15] used this technique to obtain the 3D shape of vertebrae present in the upper cervical region. The accuracy was determined as the point-to-surface distance between the reconstructed object and the reference dimensions. It was observed to be about 1 mm, which was much better than the results obtained from SCP reconstruction. Since it used more data from the radiographs, the algorithm provides more precise 3D reconstruction compared to the DLT method.

Mitulescu et al. [16] used this technique for 3D reconstruction of dried vertebrae from the lumbar region with the help of stereo-radiographs. The reconstruction error was less than 1.1 ± 1.4 mm. Pomero et al. [17] used a similar method for 3D reconstruction of thoracic and lumbar vertebrae. The 3D positions of the 25 corresponding points were obtained by this method, which contains 6 SCPs and 19 NSCPs. The validation was executed on 14 patients with 58 scoliotic vertebrae. The CT scan reconstruction was considered the ground truth. The study showed an error of 1.5 ± 2 mm using the NSCP method. Zhang et al. [18] used the NSCP method along with the epipolar geometry for the 3D reconstruction of the scoliotic spine. The search for corresponding points was reduced by restricting the search on the epipolar line. This process considerably reduces the intra and inter-observer variability.

Kumar et al. [35] also utilized this method for spine reconstruction and used it as a ground truth for comparing their results with reference to their proposed method. NSCP is the only method that gives 3D reconstruction with the highest accuracy and can be used as ground truth. This is because NSCP reconstruction is obtained with radiographs in the standing position, whereas the CT and MRI are obtained in the supine position. SCP reconstruction and NSCP reconstructions are known as point-based methods. They are highly dependent on the expert’s skills in identifying the stereo-corresponding points. These techniques need about 4–6 h for the 3D reconstruction because they need physical identification of SCPs and NSCPs [37].

### 3.3. Hybrid Methods

This classification belongs to techniques attained from the combination of various types of models from different sources. Due to their characteristics, they can be considered a distinct class called hybrid methods, though these techniques have followed procedures similar to previously stated classes. A combination of methods was tested by researchers to achieve better accuracy and morpho-realistic spine models. Kadoury et al. [38,39] proposed a hybrid image-based and statistical approach. They merged statistical knowledge and image-based characteristics from the biplanar radiographs for their hybrid 3D spine reconstruction. In this method, the centerlines of the spine are obtained from the pre-operative radiographs. These centerlines are mapped to the 3D model of the spinal curve of a scoliotic 3D spine database. By performing analytical regression, a statistical 3D modeling of the spine is achieved. Support vector regression is used to infer the 3D reconstruction of the spine. For this purpose, local linear embedding techniques are used that map 3D splines to lower dimensional space. This method needs a calculation period of 2.4 min in addition to the time required for recognizing the splines. A large database of vertebrae is needed to generate the statistical model. This statistical approach is based on local linear embedding and is sensible to insufficient sampling [20]. The average point-to-surface factual error between the reconstructed vertebra and the corresponding MRI model was 1.18 ± 0.9 mm for thoracic vertebra and 1.21 ± 1.13 mm for lumbar vertebra. This accuracy is acceptable for subjects with moderate scoliosis.

Kumar et al. [21] proposed a hybrid method using SCP reconstruction and some geometrical inferences obtained from the radiographs for 3D reconstruction of a scoliotic spine. This is called the combined SCP and geometric model (CSCPG). Initially, SCP reconstruction proposed by Kadoury et al. [32] was used for 3D spine reconstruction with the help of a calibration object. Then, geometrical inferences were extracted from the biplanar radiographs using various image processing techniques. The 3D model obtained from SCP reconstruction was fine-tuned with the help of these geometrical inferences to obtain an accurate 3D spine model. By taking the NSCP-reconstructed model as a reference, the proposed model was validated both qualitatively and quantitatively. The observed variability of CSCPG reconstruction was very low, as it required only six landmarks per vertebra to be identified. The mean reconstruction accuracy was found to be within the standard limits. Gangnet et al. [21] have proposed a study on the variability of the spine and pelvis location with respect to the gravity line. The spine reconstruction algorithms were applied to both spine and pelvis. Using a force platform and a standard stereo-radiographic method, they acquired 3D geometry of the spine and placed it with reference to the line of gravity. The accuracy was within the acceptable limits for subjects with mild and moderate scoliosis.

## 4. 3D Reconstruction from a Single Radiograph

**Figure 4:**
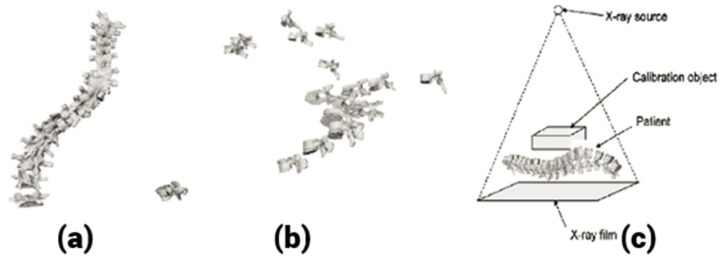
3D reconstruction of the spine from a single radiograph. (**a**) Frontal view, (**b**) lateral view, (**c**) test setup.

Novated et al. [40] presented a novel method for 3D reconstruction of a scoliotic spine from a single radiograph. The calibration of the radiograph is performed using a small object and a novel calibration algorithm. The unknown information present in the single radiograph is fulfilled by the process of registration of known 3D geometric models. Figure 4 shows this test setup. The error obtained due to the 3D/2D registration process is adjusted by the alignment constraints of individual vertebrae. The 3D models of 15 scoliotic patients were used in simulation experiments. The same number of patients was used for validation experiments, too. The experiments showed that the proposed method is accurate and robust in nature. The average RMS reconstruction error was found to be 2.89 mm, which was inappropriate for clinical applications. In addition, it requires known 3D geometric models for the reconstruction and complex calibration steps. Hence, 3D reconstruction from biplanar radiographs is found to be the most appropriate method for scoliotic spine reconstruction.

## 5. Surface Topography

In the 1970s, for the early identification of scoliosis, an early form of topographical assessment of the spine known as Moire topography [22] was used. In this technique, prototypes of shadow lines are projected onto a physical surface by projections of light beams. With the knowledge of the distance from the camera and the light source, detailed variations in the patient’s surface structure can be computed. The advantage of this technique is the ease of application and lack of radiation exposure.

**Figure 5:**
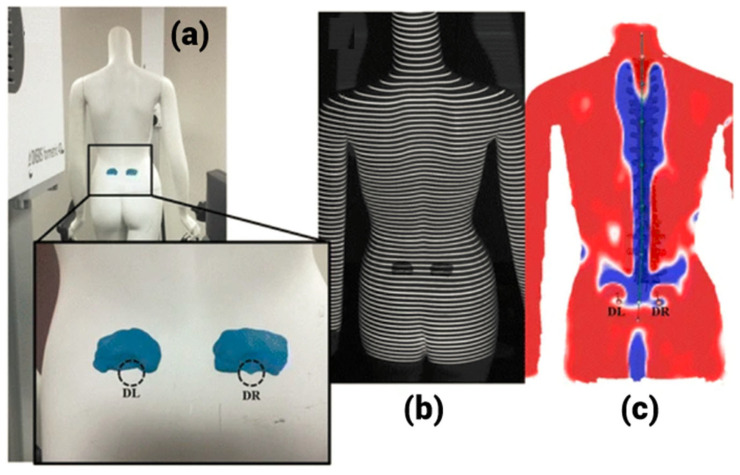
DIERS formetric 4D: surface topography scan. (**a**) The left and right sacral dimples, (**b**) images obtained by the DIERS system, (**c**) verification that dimples were precisely localized on the 3D model constructed by the DIERS system [43].

However, the limitations include variable accuracy and cost-effectiveness. Many methods that employ surface topography to quantify rotational abnormality were developed [41,42]. Drerup et al. [24] developed an imaging technique known as raster-stereography. Here, 3D reconstructions of a scoliotic spine was possible without radiation exposure. Based on this technique, the DIERS formetric four-dimensional, a non-contact scanner, was developed. In short, it is called the DIERS system, and it is capable of producing surface topography-based 3D model of the spine [43]. Figure 5 shows the DIERS system and the 3D surface topographic models.

The DIERS system can be used to evaluate spinal balance, trunk rotation, and major curves in the case of moderate to severe scoliosis. Frerich et al. [44] evaluated the DIERS system and standard radiography as a reliable option for assessing patients with adolescent idiopathic scoliosis. The correlation between the major curves in sixty-four subjects was calculated using the DIERS system and the standard radiographic technique was high. The correlation coefficients of 0.87 and 0.76 for thoracic and lumbar vertebrae, respectively, were observed in this case. A reproducibility test of thirty repetitive DIERS system measurements was conducted on fourteen subjects. It was found to be large, with a coefficient of reliability of up to 0.997. Hence, the DIERS system was proven to be equivalent to the standard radiographic technique.

Knott et al. [45] compared the reproducibility and reliability of the DIERS system with respect to standard radiographs. One hundred ninety-three pediatric subjects with adolescent idiopathic scoliosis underwent standing radiographs and surface topography scans with the DIERS system. The reproducibility of the lumbar curve, thoracic curve, and thoracic kyphosis proportions using the DIERS system topography was very high, and the correlation coefficient ranged from 0.86 to 0.94. The correlation between the standard radiographic measurements and the DIERS system measurements was also high for various curves of the spine. With these potential results, the researchers proposed that the DIERS system produces topographic scans that are equivalent to radiographic scans. Hence, harmful radiation can be completely eliminated in the case of scoliotic screening.

In more recent studies, Tabard-Fougere et al. [46] compared the reliability of the DIERS system with low-dose radiographic systems in subjects with adolescent idiopathic scoliosis. Quantification results obtained from thirty-five subjects show a high correlation among these methods. There were no major difference between the major curve using the low-dose radiographic system and the DIERS system. The inter- and intra-observer dependability were also outstanding. These studies confirm the use of surface topographic methods as a noninvasive alternative for monitoring curve progression in adolescent scoliosis. This method has the capability to reduce the use of radiographs, which in turn decreases both cost and radiation exposure for the subject.

Komeili et al. [47] utilized a VIVID 910 3D-laser digital scanner to evaluate the dependability of measuring torso asymmetry using the surface topography method in forty-six patients with adolescent idiopathic scoliosis. The inter-observer variability was outstanding. The intra-observer and test–retest reliability were also similar to the DIERS system. This innovative surface topography method was found to be a promising noninvasive tool for evaluating scoliotic deformity progression [48]. Thus, the surface topography method can be used as a radiation-free alternative for spinal alignment. The constraints include the initial cost burden and the underestimation of the spinal curvature compared with radiographs. The measures from surface topography are comparable to radiographs and are independent of the operator’s influence [49].

## 6. Low-Dose Radiography

**Figure 6:**
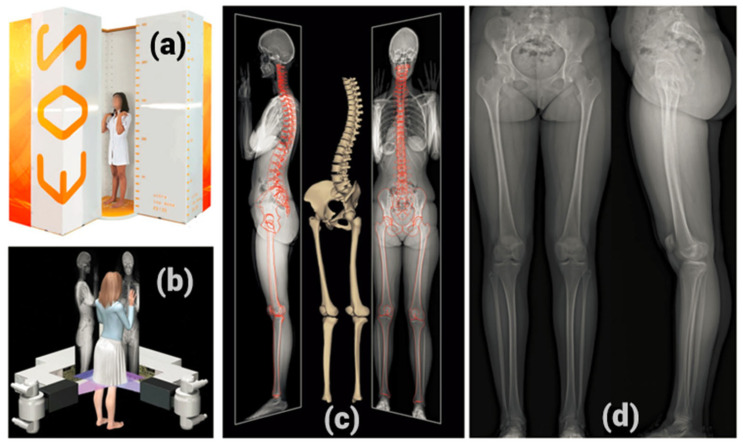
EOS imaging system. (**a**) Patient positioning during exam, (**b**) basic operation, (**c**) 3D modelling of the spine and lower limb, (**d**) example of typical EOS lower-limb pair [52].

The concept of low-dose radiography came into existence after the year 1992. Multiwire proportional chambers were developed, which led to a Nobel prize in physics. These can be used to generate diagnostic radiographic images with low radiation dosage [50]. It is a vertical biplanar slot-scanning technology. The low-dose radiographs can produce fine-quality images with 60% to 80% less radiation than conventional radiographs [51,52]. The system of low-dose radiography that is popular in scoliotic spine imaging, is the EOS imaging system. It comprises a pair of X-ray tubes and orthogonally placed detectors. This permits synchronized acquisition of posterior–anterior and lateral radiographs in the standing position. Figure 6 shows the EOS imaging system and its sample radiographs.

This method lowers the radiation dose required to obtain a 2D radiograph of the spine by eight to ten times [53]. Hirsch et al. [54] compared standing and bending EOS images with conventional bending radiographs to evaluate subjects’ preoperative conditions. No substantial differences were found in the major curves between the EOS imaging and normal radiographs in the thoracic and lumbar regions. They also performed analysis on Lenke curves, which indicated no significant differences in the spinal dimensions between EOS and normal radiographs. It had further advantages of reduced exposure of radiation and ease in obtaining radiographic images for the subject as well as the technician.

Illés et al. [27] assessed EOS imaging in subjects with adolescent idiopathic scoliosis experiencing surgical alteration. They related it with measurements produced from 2D EOS imaging. Ninety-five subjects were included in this study. The pre- and postoperative examinations were performed to match the correlation between conventional EOS 2D measurements and 3D reconstructions. A preoperative correlation coefficient of 0.95 and postoperative correlation coefficient of 0.93 resulted from this study. This study proved that EOS imaging can be the best alternative to existing 3D reconstruction methods. Studies conducted by Somoskeoy et al. [55] and Sangole et al. [56] proved the precision and accuracy of the EOS imaging system with reference to the gold standard 3D spine reconstruction techniques.

EOS imaging has been proven to be as efficient in the 3D evaluation of deformities like scoliosis as conventional 2D assessment with standard radiographs. It has an added advantage of reduced radiation exposure. EOS imaging technology can characterize the deformity in 3D in the sagittal, coronal, and axial planes. However, the initial investment required to implement this imaging technology is very high. In addition, it has limited accessibility and decreased cost-effectiveness compared to all the existing methods. In order to obtain the 3D model of the spine, a full-body radiograph is necessary, which is another drawback of this system.

## 7. Ultrasonography

It is a non-ionizing imaging system that provides real-time image findings at a very low cost. Spinal ultrasonography is built on the theory that the laminae and spinous processes are precise landmarks for evaluating 3D spinal deformations [57]. The vertebral turning can be verified by the rotation of the laminae with reference to the leaning of the ultrasound sensor. Suzuki et al. [58] first demonstrated the use of ultrasonography in spinal imaging. Chen et al. [59] proved that the center-of-lamina method to measure curve magnitude and rotation of the vertebra was equivalent to the conventional measurements achieved through radiographs. Brink et al. [60] examined the validity and reliability of ultrasonography for computing spinal deformity in thirty-three subjects with adolescent idiopathic scoliosis. They employed the transverse processes and spinous processes as markers. In this study, the correlations between the major curves and ultrasound angles were outstanding.

Young et al. [61] studied the effectiveness of ultrasonography to identify curve advancement in idiopathic scoliosis. The major curves were estimated using the center-of-lamina technique, and the consistency of ultrasonography for computing spinal curves was compared between observers with or without radiographs. A very high sensitivity and specificity was observed for the ultrasonography for detecting curves. Despite these studies, ultrasonography had the following limitations. Zheng et al. [62] examined the operator-dependent characteristics of ultrasonography. The qualified operator got fewer curves with a large discrepancy between the ultrasound and radiographs compared with those performed by the trainees. This demonstrated the high observer variability with this modality. The use of ultrasonography to find scoliotic curves was limited to subjects with mild to moderate scoliosis with major curves of less than 45 degrees [63]. Lee et al. [49] conducted a study in which they obtained promising results for patients with adolescent idiopathic scoliosis with different severity curves. They found that these curves can be evaluated and monitored by ultrasound imaging, reducing the usage of radiation during follow-ups. This method could also be used for scoliosis screening. Tin et al. [63] conducted a study in which they proved that ultrasonography can be used for evaluating the effectiveness of nonsurgical treatments.

Ultrasonography is an easily available modality and has the advantages of no radiation and low cost. The limitations include limited detection of lower-degree curves and the larger possibility for human error. However, it can be used to securely examine curve advancement over time without the requirement for frequent short-interval radiographic measurements.

## 8. Discussion and Conclusions

Three-dimensional reconstruction of the human spine from multiplanar radiographs has limitations such as a risk of high radiation dosage and non-availability of standard image formats. Hence, biplanar radiographs are used for 3D reconstruction, which are readily available from follow-up examinations. However, these techniques strongly depend on the operator skills. It is tough to precisely recognize and match points on biplanar radiographs. Thus, they cannot guarantee reproducibility. In addition, they cannot be used for structures like bones due to the lack of anatomical landmarks. Furthermore, manual detection of anatomical landmarks is a complex process and time-consuming. Hence, these methods cannot be applied during normal clinical practice. Regardless of these drawbacks, SCP-based techniques still are used for 3D modelling because of the simplicity of their procedures. The precision of these techniques is close to CT scan results and clinically acceptable in nature. The hybrid techniques can have distinct properties with reference to their combinations. These techniques can increase the outcomes if they are consistent with the purpose. Nonetheless, they have constraints due to their combination of techniques. Three-dimensional reconstruction from a single radiograph requires known 3D geometric models for the reconstruction. It involves complex calibration steps, and the accuracy is poor.

In order to minimize the risk of radiation exposure, new modalities have been developed, including surface topography, low-dose radiography, and ultrasonography. Surface topography and ultrasonography offer the advantage of completely removing radiation exposure. However, they are less precise in quantifying the spinal curvature. In future, the limitations of ultrasonography and surface topography in computing the exact spinal curvatures should be addressed. The surface topography and ultrasonography systems are economical compared to the EOS imaging system. Present studies have proven the benefit of previous radiographs as a baseline for ultrasonography and surface topography. Future directions must focus on the development of uniform protocols for reducing the overall radiation exposure. Surface topography cannot assess real bone morphology in the same way as a radiograph. If surface topography can provide reliable and comparable results, it should replace radiographs. Therefore, researchers should work towards developing surface topography techniques that can produce accurate scoliotic curves during diagnosis and follow-up examinations. Though ultrasonography can be used for 3D reconstruction of scoliotic spines, a reliable and valid 3D ultrasound system ready for clinical scoliosis assessment has not yet been reported. Low-dose radiography, namely, EOS imaging, was found to yield a very accurate 3D reconstruction of the spine. Though it has decreased radiation exposure, it requires a significant initial investment. Also, it takes a full-body scan of the subject, which is not necessarily required for 3D spine reconstruction. Future directions should focus on low-cost EOS imaging equipment and related software. This will certainly decrease the use of ionizing radiation in diagnosis and follow-up examinations for scoliosis. The 3D reconstruction of scoliotic spine can also be used in planning surgery, biomechanical applications, and computer-assisted surgery.

## Data Availability

Not applicable.
